# Efficacy and safety of a novel 450 nm blue diode laser versus plasmakinetic electrocautery for the transurethral resection of non-muscle invasive bladder cancer: The protocol and result of a multicenter randomized controlled trial

**DOI:** 10.3389/fonc.2022.1065735

**Published:** 2023-01-17

**Authors:** Kaijie Wu, Dali Jiang, Lianhua Zhang, Shuai Jiang, Tianhai Lin, Yi Luo, Jinhai Fan, Tao Yang, Haige Chen, Peng Zhang, Xinghuan Wang, Qiang Wei, Jianming Guo, Yiran Huang, Dalin He

**Affiliations:** ^1^ Department of Urology, The First Affiliated Hospital of Xi’an Jiaotong University, Xi’an, China; ^2^ Department of Urology, Renji Hospital Affiliated to Shanghai Jiaotong University School of Medicine, Shanghai, China; ^3^ Department of Urology, Zhongshan Hospital, Fudan University, Shanghai, China; ^4^ Department of Urology, West China Hospital, Sichuan University, Chengdu, China; ^5^ Department of Urology, Zhongnan Hospital of Wuhan University, Wuhan, China

**Keywords:** blue diode laser, plasmakinetic electrocautery, TURBt, NMIBC, RCT

## Abstract

**Objectives:**

To be the first to apply a novel 450 nm blue diode laser in transurethral resection of bladder tumor (TURBt) to treat patients with non-muscle invasive bladder cancer (NMIBC) and evaluate its efficacy and safety during the preoperative period compared to the conventional plasmakinetic electrocautery.

**Materials and Methods:**

Randomized controlled trial (RCT) in five medical centers was designed as a non-inferiority study and conducted from October 2018 to December 2019. Patients with NMIBC were randomized to the blue laser or plasmakinetic electrocautery group for TURBt. As the first study to evaluate this novel blue laser device, the primary outcome was the effective resection rate of bladder tumors, including effective dissection and hemostasis. The secondary outcomes were the perioperative records, including surgical time, postoperative indwelling catheter time, hospital stay length, blood loss, reoperation rate, wound healing and adverse events.

**Results:**

A total of 174 patients were randomized to either the blue laser group (85 patients) or plasmakinetic electrocautery group (89 patients). There was no statistical significance in the clinical features of bladder tumors, including tumor site, number and maximum lesion size. Both the blue laser and plasmakinetic electrocautery could effectively dissect all visible bladder tumors. The surgical time for patients in the blue laser group was longer (p=0.001), but their blood loss was less than that of patients in the control group (p=0.003). There were no differences in the postoperative indwelling catheter time, hospital stay length, reoperation rate or other adverse events. However, the patients undergoing TURBt with the blue laser showed a faster wound healing at 3 months after operation.

**Conclusion:**

The novel blue laser could be effectively and safely used for TURBt in patients with NMIBC, and this method was not inferior to plasmakinetic electrocautery during the perioperative period. However, TURBt with the blue laser may provide the benefit to reduce preoperative blood loss and accelerate postoperative wound healing. Moreover, longer follow-up to confirm recurrence-free survival benefit was required.

## Introduction

Bladder cancer is the 10th most common type of cancer in the world, and approximately 75% of newly diagnosed bladder cancers are non-muscle invasive bladder cancers (NMIBCs), including Ta, Tis and T1 (1, 2). Transurethral resection of bladder tumor (TURBt) using different medical devices, such as monopolar or plasmakinetic electrocautery, lasers and HybridKnife, is recommended for the initial surgical treatment of NMIBC (3–5).

Until now, different kinds of lasers, including holmium lasers, thulium lasers and potassium titanyl phosphate (KTP) green lasers, have been used in TURBt (6, 7). The application of these lasers could overcome the shortcomings of conventional TURBt, such as high amounts of bleeding, obturator nerve reflex and bladder perforation. Additionally, these lasers could be used to achieve en bloc resection of tumors, which could provide high-quality postoperative specimens for accurate pathological diagnosis (8–10). In our previous study, we have demonstrated that en bloc resection improves the identification of muscularis mucosae in NMIBC, which may enhance the accurate identification of the T1 substage (11, 12).

The 450 nm wavelength blue diode laser is a novel laser for the vaporization, ablation and coagulation of different soft tissues (13–15), and its power is stronger than these available lasers, such as holmium and green laser. Commercially, several innovative medical instruments based on a high-power and high-efficiency blue light-emitting diode (LED) have been approved for surgery in the Department of Stomatology or Otorhinolaryngology. In our previous study, we demonstrated that a 30 watt blue diode laser, similar to the 532 nm green laser, is capable of producing highly efficient bladder tissue vaporization and coagulation, and results in low penetration and low thermal damage to adjacent tissues ex vivo and *in vivo (* 13). Therefore, the blue laser may be a new and safe alternative for surgeries to perform on patients with superficial bladder diseases. Based on this premise, we conducted a non-inferiority study to assess the efficacy and safety of 450 nm blue diode laser device for the transurethral resection of NMIBC, as compared to conventional plasmakinetic electrocautery.

## Materials and methods

### Study design and participants

This randomized controlled trial (RCT) was designed by urologists from the First Affiliated Hospital of Xi’an Jiaotong University (Xi’an, P.R. China) and Renji Hospital Affiliated to Shanghai Jiaotong University School of Medicine (Shanghai, P.R. China). The trial protocol and informed consent form were approved by the ethics committees of five centres (First Affiliated Hospital of Xi’an Jiaotong University, Renji Hospital Affiliated to Shanghai Jiaotong University School of Medicine, Zhongshan Hospital of Fudan University, Shanghai, West China Hospital of Sichuan University, and Zhongnan Hospital of Wuhan University) with trial registration in Chinese Clinical Trial Registry. A total of 180 eligible patients with suspicious primary or recurrent, benign or malignant NMIBC were recruited based on cystoscopic examination and pelvic CT scan before surgery between October 2018 and December 2019 using the following inclusion criteria: adults aged 18–80 years, an Eastern Cooperative Oncology Group (ECOG) score of 0-1 and suitable for endoscopic treatment. Patients who were not suitable or refused to undergo surgical treatment were excluded. In addition, we excluded patients with tumors size ≥2 cm or number ≥4 because these tumours were difficult to extract without causing damage or required much more surgical time based on our previous study (7).

### Randomization and grouping

Patients were randomly assigned in a 1:1 ratio to receive TURBt by the blue laser or plasmakinetic electrocautery. An independent statistician used computer-generated random numbers to create the random number list before the beginning of the study. Only investigators (surgeons) knew the group and applied the indicated medical devices, but others were blinded. After a clinical assessment to determine whether patients met the inclusion and exclusion criteria, all patients needed to provide written informed consent before being enrolled and undergoing surgery. Then, randomization was performed by the investigators according to the random number list.

### Treatment procedures

All patients were treated with the same management apart from their form of surgery by either the conventional plasmakinetic electrocautery or blue laser surgery. To reduce surgical heterogeneity, all procedures were performed by the surgeons who had the most expertise and undergone the same training. Conventional TURBt was performed with the plasmakinetic loops (Olympus, Japan). The standard procedure of the blue laser for TURBt in patients with NMIBC was the same as that of other lasers in our previous experience (7). Briefly, this blue laser equipment was a novel 450 nm high-power blue diode laser with a front-firing fiber (Blueraymed, Xi’an, China), and a modified technique using a front-firing fiber to en bloc enucleate bladder tumors was applied (7). The laser energy was 0.3 J×50 Hz (15 W) during surgery. Tumor specimens were processed and reviewed by pathologists who were blinded to patients’ clinical information and surgery type. Postoperative management (when to remove the urinary catheter and discharge the patients) was performed by the doctor who was also blinded to the surgical information of the patients. After the operation, all the patients underwent intravesical instillation and follow-up according to the recommendation of the guidelines. All the patients with high-risk NMIBC will undergo re-TURBt in this study.

### Clinical outcomes

The primary outcome was the effective resection rate of bladder tumours, including effective dissection and hemostasis. The secondary outcomes were the perioperative records, including surgical time, postoperative indwelling catheter time, hospital stay length, blood loss, reoperation rate and adverse events. Hemoglobin was assessed 24 h after surgery. Additionally, the wound healing and tumor recurrence at 3 months after operation were compared between two groups by another surgeon who was blinded to patients’ allocation. The relative wound healing rate (%) was calculated by the proportion of wounds covered by normal urothelial epithelium to the whole original wounds. Tumor stage was assessed based on the 2017 TNM classification of urinary bladder cancer, and tumor grade was based on the 2014 WHO grading classification. Blood loss was estimated based on the hemoglobin difference before and after surgery. Since the main purpose of this study was to evaluate the application of blue lasers for the transurethral resection of tumours in patients with NMIBC, long-term oncological outcomes and patient survival were not reported here.

### Statistical analysis

Sample size calculations were performed using PASS software (2011, NCSS, LLC, Kaysville, Utah, USA) for this randomized controlled trial. Based on the results of the previous studies, the efficiency of TURBt in tumor resection was more than 97%. We assumed that the blue laser was not inferior to plasmakinetic electrocautery in tumor resection; in addition, the power was 0.80, the significance level was 0.05, the noninferiority margin (Δ) was 0.1, and the exclusion rate was 20%. Finally, it was determined that data from at least 90 patients per arm of the study were required. Categorical data were presented as frequencies, and continuous data were presented as the mean ± standard deviation (SD) or median (interquartile range, IQR) based on the characteristics of the data. Between-group differences were assessed with the chi-square test or Fisher’s exact test for categorical variables and Student’s t test or Mann-Whitney *U* test for all continuous measures. All statistical tests were two-tailed with an α of 0.05. Data were analyzed with SPSS version 24 (SPSS Inc., Chicago, IL, USA).

## Results

**Figure 1:**
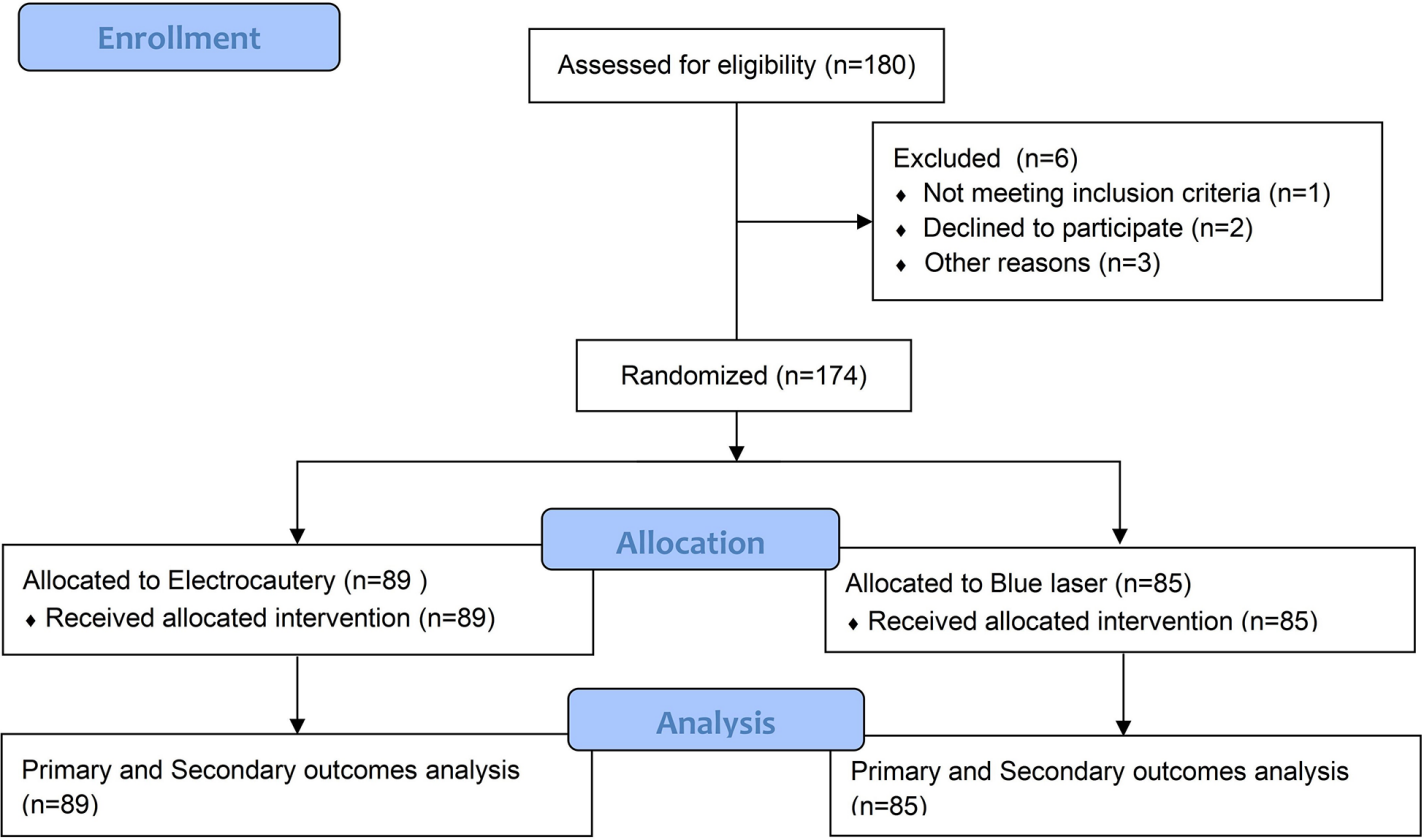
Patient selection process and exclusions.

Between October 2018 and December 2019, 174 patients were enrolled, of whom 89 were randomly assigned to undergo plasmakinetic electrocautery and 85 were designated to undergo blue laser for TURBt (Flow diagram shown in **Figure 1**). The baseline characteristics of the patients in two groups in terms of age, BMI, nationality, tumor size, number and location were well balanced (**Table 1**), but the difference in sex distribution was marginally significant (*p*=0.048).

**Table 1:** Baseline characteristics of the 174 patients included in the study.

Characteristics	Electrocautery (n=89)	Blue laser (n=85)	P value
Age (yr),			0.912
mean ( **±** SD)	59.10 ± 12.26	59.31 ± 12.03	
Sex, n (%)			0.048
Men	67 (75.3)	74 (87.1)	
Women	22 (24.7)	11 (12.9)	
BMI (kg/m^2^), mean ( **±** SD)	23.92 ± 3.52	24.20 ± 3.17	0.575
Nationality, n (%)			0.489^#^
Han	89 (100.0)	84 (98.8)	
Other	0 (0.0)	1 (1.2)	
Tumour size (cm)			0.210
mean ( **±** SD)	1.31 ± 0.57	1.43 ± 0.64	
Tumour number, n (%)			0.381^*^
median (min, max)	1 (1-3)	1 (1-3)	
Tumour location, n (%)			0.660
Anterior wall	3 (2.3)	6 (5.6)	
Posterior wall	11 (8.3)	14 (13.1)	
Lateral wall	79 (59.8)	58 (54.2)	
Trigone	8 (6.1)	14 (13.1)	
Bladder neck	15 (11.4)	9 (8.4)	
Other	16 (12.1)	10 (9.3)	

BMI, body mass index; SD, standard deviation; *Wilcoxon rank sum test; ^#^Fisher exact test.

Based on the parameters of effective dissection and hemostasis, both the blue laser and plasmakinetic bipolar current could be used to completely dissect all visible bladder tumours. Therefore, the effective resection rate of bladder tumours in two groups was 100% (**Table 2**), and the 95% confidence interval was greater than -10%, indicating that tumor resection by the blue laser was not inferior to plasmakinetic electrocautery. Additionally, blood loss was significantly less in patients in the blue laser group than in patients in the plasmakinetic electrocautery group (p=0.002; **Table 2**). According to the Clavien-Dindo classification, one subject in the blue laser group required a second surgery to stop active intravesical bleeding and evacuate blood clot (Clavien-Dindo IIIb). No blood transfusion, prolong irrigation and hospital stay were observed due to blood loss in this clinical trial.

**Table 2:** Perioperative and postoperative pathological characteristics of patients.

Characteristics	Electrocautery (n=89)	Blue laser (n=85)	P value
Effective resection rate, **n (%)**	89 (100.0)	85 (100.0)	–
Surgical time (min), mean ( **±** SD)	29.3 ± 18.3	40.3 ± 25.6	0.001
Catheter time (d), mean ( **±** SD)	3.07 ± 3.08	3.81 ± 3.66	0.154
Postoperative hospital stay length (d), mean ( **±** SD)	2.29 ± 1.22	2.39 ± 1.40	0.629
Hb decrease (g/L), mean ( ± SD)	7.00 ± 8.10	6.77 ± 10.10	0.002
Obturator nerve reflex, n (%)	3 (3.33)	0 (0.00)	0.246
Bladder perforation, n (%)	0 (0.00)	1 (1.11)	0.489
Tumour stage, n (%)			0.716
Ta	43 (48.3)	36 (42.4)	
T1	31 (34.8)	32 (37.6)	
Other	15 (16.9)	17 (20.0)	
Tumour grade, n (%)			0.588
Low grade	41 (45.6)	42 (49.4)	
High grade	29 (32.6)	30 (35.3)	
Other	19 (21.4)	13 (15.3)	

^#^Fisher exact test. Hb, haemoglobin. Other, benign tumours.

However, the surgical time was longer in patients in the blue laser group than in patients in the plasmakinetic electrocautery group (p=0.001; **Table 2**). The postoperative hospital stay and catheterization time were not different between these two groups of patients. Overall, the catheterization time and hospital stay seem to be prolonged in both groups, because day surgery was still not popular in China and several subjects were discharged with urinary catheter due to bladder perforation or urethral stricture. Regarding the complications and postoperative pathological results, three patients (3.33%) had an obturator nerve reflex during TURBt, but none of the patients in the blue laser group had an obturator nerve reflex. Due to the limitation of operability by laser in the anterior wall of the bladder, one patient in the blue laser group experienced bladder perforation and immediately transferred to routine electrocautery TURBt. Additionally, there was no difference in tumor grade or stage and the presence of detrusor muscle based on the pathological results between two groups.

**Figure 2:**
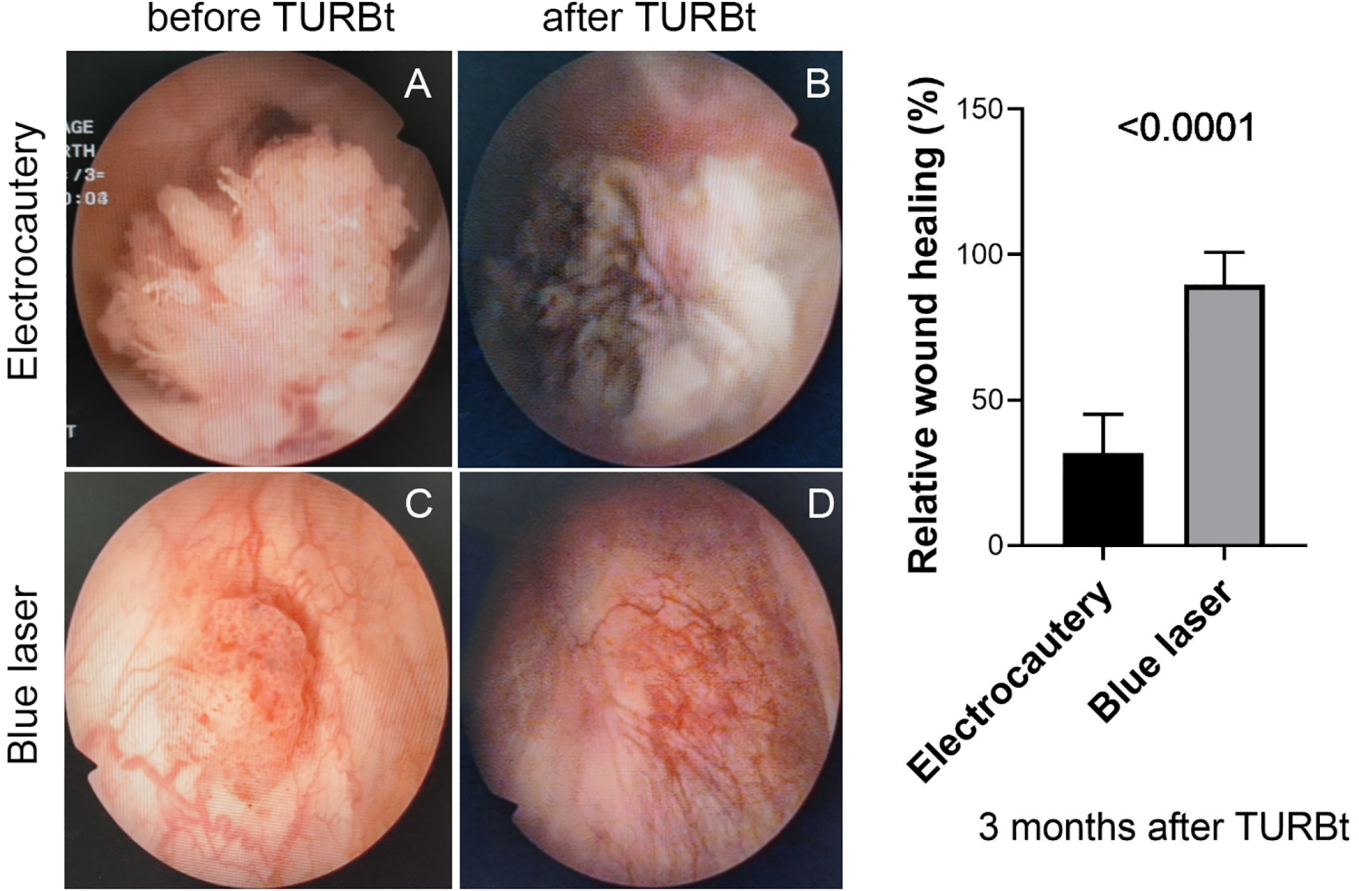
Cystoscopic examination of bladder tumours before TURBt and wound healing at 3 months after TURBt. Left panel, two representative patients in different groups treated with plasmakinetic electrocautery (**A**, before TURBt; **B**, after TURBt) or blue laser (**C**, before TURBt; **D**, after TURBt). Right panel, statistical analysis of wound healing rate between two groups after TURBt with plasmakinetic electrocautery or blue laser, P<0.0001.

Also, the wound healing and tumor recurrence at 3 months after operation were compared between two groups. No tumor recurrence was observed in both groups, but a faster wound healing was observed in patients in the blue laser group. The wound was almost completely covered by the urothelial mucosa in the blue laser group at 3 months after TURBt, however, the wound scar could be obviously observed in most of patients by plasmakinetic electrocautery (relative wound healing rate 89.7% vs. 31.8%, P<0.0001, **Figure 2**).

## Discussion

Piecemeal or en bloc resection using monopolar or plasmakinetic electrocautery, Thulium-YAG, Holmium-YAG or Greenlight-KTP laser and HybridKnife is feasible in the diagnosis and treatment of TaT1 bladder tumours (5, 6, 8). In particular, in patients with a history of small, Ta-LG/G1 tumours, laser vaporization or fulguration of small papillary recurrences on an outpatient basis can reduce the therapeutic burden (1). However, most of these laser equipment are very large, noisy and expensive and requires a special power supply. Therefore, new laser devices with simple industrial manufacturing and low cost are required for developing countries.

Blue diode lasers have received more attention since Shuji Nakamura invented a high-power and high-efficiency blue LED and won the Nobel Prize for Physics in 2014 (16). Compared to other visible or infrared lasers with different wavelengths, 450 nm blue lasers have relatively shorter wavelengths and higher absorption in soft tissues, such as hemoglobin and melanin. When this laser is absorbed by soft tissues, ablation and coagulation of tissues and blood vessels may occur, leading to less bleeding during surgery (13–15). Additionally, the penetration depth of blue light is much shallower than that of red light, which leads to fewer accidental injuries in deeper tissue layers. In our previous study, we demonstrated that a 30 watt blue diode laser, similar to a 532 nm green laser, is capable of producing highly efficient bladder tissue vaporization and coagulation, and results in low penetration and low thermal damage to adjacent tissues ex vivo and *in vivo*.

To further explore the feasibility of blue lasers in transurethral resection of superficial bladder tumours as a new and safe tool, we conducted a multicenter, randomized controlled trial to compare blue lasers with conventional plasmakinetic bipolar current in NMIBC surgery. Since the blue laser has never been clinically approved for the surgery of bladder tumors, so we design to compare en bloc bule laser resection with standard bipolar resection. For front-firing blue laser, it was much more convenient to perform en bloc resection. Indeed, both blue laser and plasmakinetic bipolar current could effectively dissect all visible bladder tumours, which indicated that different tools could be used for tumor dissection. Surgery with a blue laser required more time but resulted in less blood loss. The reason was that a blue laser was suitable for en bloc tumor enucleation, and more time was required if the investigators chose this technique. However, the blue laser could provide a good hemostasis during tissue vaporization and dissection and led to less blood loss during operation. Additionally, the lack of an obturator nerve reflex was another advantage of the use of blue lasers in the initial treatment of NMIBC. Unfortunately, due to the limitation of patient number, we did not observe the difference in tumor grade or stage between two groups. However, the patients undergoing TURBt with the blue laser showed a faster would healing at 3 months after operation, confirming its low penetration and low thermal damage to adjacent tissues. This result indicated its potential benefit to reduce the risk of bladder scar and contracture after TURBt. Therefore, this study provide the evidence to support blue laser as a new choice of a proper laser in the treatment of NMIBC (17, 18).

Taken together, in this multicenter RCT clinical trial, we demonstrated that the novel blue laser could be effectively and safely used to treat patients with NMIBC by transurethral resection, which was not inferior to plasmakinetic electrocautery. Additionally, this laser offers the advantages of less bleeding, the absence of an obturator nerve reflex and a faster would healing after operation. However, this trial had several limitations. First, different surgeons were involved, and the surgical experience might affect their treatment effects. Second, no long-term follow-up was performed to compare tumor recurrence between two groups, many patients with benign bladder tumours (i.e., papilloma and PUNLMP) were also included in this study. Third, only patients with small and fewer bladder tumours were included, which might not fully reveal the difference in treatment strategy using these two surgical devices.

## Data availability statement

The raw data supporting the conclusions of this article will be made available by the authors, without undue reservation.

## Ethics statement

The studies involving human participants were reviewed and approved by First Affiliated Hospital of Xi’an Jiaotong University, Renji Hospital Affiliated to Shanghai Jiaotong University School of Medicine, Zhongshan Hospital of Fudan University, Shanghai, West China Hospital of Sichuan University, and Zhongnan Hospital of Wuhan University. The patients/participants provided their written informed consent to participate in this study. Written informed consent was obtained from the individual(s) for the publication of any potentially identifiable images or data included in this article.

## Author contributions

Data acquisition, data analysis, and manuscript drafting: KW and DJ. Data acquisition and interpretation: LZ, SJ, TL, YL, JF, TY, HC, PZ, XW, QW and JG. Designing the research and critically revising important knowledge content and final approval of the version for publication: YH and DH. All authors contributed to the article and approved the submitted version.
